# Facile Preparation of Hydrophobic PLA/PBE Micro-Nanofiber Fabrics via the Melt-Blown Process for High-Efficacy Oil/Water Separation

**DOI:** 10.3390/polym14091667

**Published:** 2022-04-20

**Authors:** Han Li, Heng Zhang, Jun-Jie Hu, Guo-Feng Wang, Jing-Qiang Cui, Yi-Feng Zhang, Qi Zhen

**Affiliations:** 1 School of Textile, Zhongyuan University of Technology, Zhengzhou 451191, China; han300030@163.com (H.L.); zckh@sina.com (Y.-F.Z.); 2 Henan Key Laboratory of Medical Polymer Materials Technology and Application, No. 1 Yangze Road, Xinxiang 453400, China; chemwgf@163.com (G.-F.W.); ziyu_best@163.com (J.-Q.C.); zhenqi7721@126.com (Q.Z.); 3 Shanghai Earntz Nonwoven Co., Ltd., No. 88, Jiangong Road, Jinshan District, Shanghai 201501, China; jhu@shearntz.com; 4 Henan Tuoren Medical Device Co., Ltd., Tuoren Industrial Zone, No. 1 Yangze Road, Xinxiang 453400, China; 5 School of Clothing, Zhongyuan University of Technology, No. 1 Huaihe Road, Zhengzhou 451191, China

**Keywords:** oil pollution, oil/water separation, micro-nanofiber fabrics, melt-blown, polylactic acid, nonwoven, toughening

## Abstract

Polylactic acid (PLA) micro-nanofiber fabrics with a large specific surface area and excellent biodegradability are commonly used in oil/water separation; however, challenges remain due to their poor mechanical properties. Herein, a thermoplastic polylactic acid/propylene-based elastomer (PLA/PBE) polymer was prepared by blending PLA with PBE. Then, PLA/PBE micro-nanofiber fabrics were successfully prepared using a melt-blown process. The results show that the PLA/PBE micro-nanofiber fabric has a three-dimensional porous structure, improving the thermal stability and fluidity of the PLA/PBE blended polymers. The PLA/PBE micro-nanofiber fabric demonstrated a significantly reduced average fiber diameter and an enhanced breaking strength. Moreover, the water contact angle of the prepared samples is 134°, which suggests a hydrophobic capacity. The oil absorption rate of the fabric can reach 10.34, demonstrating excellent oil/water separation performance. The successful preparation of PLA/PBE micro-nanofiber fabrics using our new method paves the way for the large-scale production of promising candidates for high-efficacy oil/water separation applications.

## 1. Introduction

Over the past century, petroleum has provided power for social development and human progress; however, its extraction and transportation generate oil pollution, which is difficult to avoid [1,2,3]. Thus, oil pollution control has become an important topic. Nowadays, a series of strategies involving products such as fabrics [4,5], membranes [6], and foams [7], are used to address this problem. Among them, fabrics represent a low-cost, durable, and light-weight method [8,9,10]. However, conventional fabrics consisting of coarse fibers have poor oil/water separation properties due to their insufficient surface roughness and poor oil absorption rates [11,12].

To overcome this, nonwoven processing is used to produce a range of micro-nanofibrous fabrics, for example, electrostatic spinning [13], melt blowing [14,15], and flash spinning [16]. Electrostatic spinning is a versatile method for manufacturing hydrophobic nanomaterials while controlling their composition and structure [17,18]. Du et al. [19] prepared nanoparticle co-blended nanofiber fabrics and found that porous micro-nanofibrous fabrics with a graded, rough structure exhibited good separation efficiency for different emulsions. Meng et al. [20] prepared hydrophilic polyvinyl pyrrolidone (PVP)/polyacrylonitrile (PAN) nanofiber fabrics from electrospinning fibers to separate oil/water mixtures under harsh conditions using gravity. However, electrostatic spinning is not sufficiently productive, and the equipment is complex and difficult to shape with thermoplastic polymers [21,22]. In contrast, the melt-blown process effectively produces large-scale functional micro-nanofibrous fabrics at a high production efficiency, with a low pollution output, in a simple process [23]. Zhang et al. [24] prepared hydrophobic lipophilic polypropylene (PP) micro-nanofibrous fabrics with a significant overall roughness using a blending melt-blown process. These micro-nanofibrous fabrics can easily separate oils from various aqueous solutions with a high separation efficiency. Sun et al. [25] prepared hydrophobic polypropylene/titanium dioxide micro-nanofibrous fabrics via a melt-blown process. These fabrics exhibited a stable oil/water separation performance and were produced on a large scale. However, as a petroleum-based polymer, PP causes secondary pollution due to the time it takes to degrade (hundreds of years) [26]. As an alternative, polylactic acid (PLA) is a sustainable polymer that uses corn [27] as the primary material and has good biodegradability [28]. Nevertheless, as a result of its limited mechanical properties, such as poor extensibility [29], PLA micro-nanofibrous fabrics are still in the blending modification research stage. Propylene-based elastomer (PBE) is an elastic polymer that improves the toughness of PLA [30,31,32]. Wang et al. [33] investigated its mechanical properties and, through morphological analyses of PP/PBE blends, explored the toughening mechanism of PBE. The results showed that PBE could effectively improve the impact strength of PP/PBE blends by adding rubber compositions. Krishnan et al. [34] prepared PLA/PBE blends by melt-blending PBE elastomers with PLA and investigated the mechanical properties and morphological structures of the PBE blends. These results show that PBE improves the breaking strength and elongation properties [31,35,36]. However, to the best of our knowledge, there are few studies that focus on PLA/PBE micro-nanofiber fabrics for high-efficacy oil/water separation.

In this work, we propose a PLA/PBE micro-nanofiber fabric prepared using melt-blown technology from the raw materials of blended PLA and PBE polymers. The thermal and rheological properties of PLA/PBE blended polymers were investigated, and the morphology, mechanical properties, and oil/water separation properties were analyzed.

## 2. Materials and Methods

### 2.1. Materials

PLA chips (6252D, Nature Works LLC, Minnetonka, MN, USA) with a melt flow index of 21.3 g/10 min at 210 °C and a density of 124 g/cm^3^, and PBE chips (7050 L, Exxon Mobil Corporation, Irving, TX, USA) with a melt flow index of 48.5 g/(10 min) and a density of 0.865 g/cm^3^ was used. PLA chips were dried in a vacuum-drying oven (DHG-9076A, Shanghai Jinghong Experimental Equipment Co., Ltd., Jinghong, China) at a temperature of 80 °C for 12 h. The dried PLA chips were mixed with PBE chips in a high-speed mixer (DS-PWD-MIXER, Nanjing Black Magnetic Machinery Co., Ltd., Nanjing, China, 100 r/min, 30 min) with PBE mass ratios of 5%, 10%, 15%, and 20%.

### 2.2. Preparation

**Figure 1:**
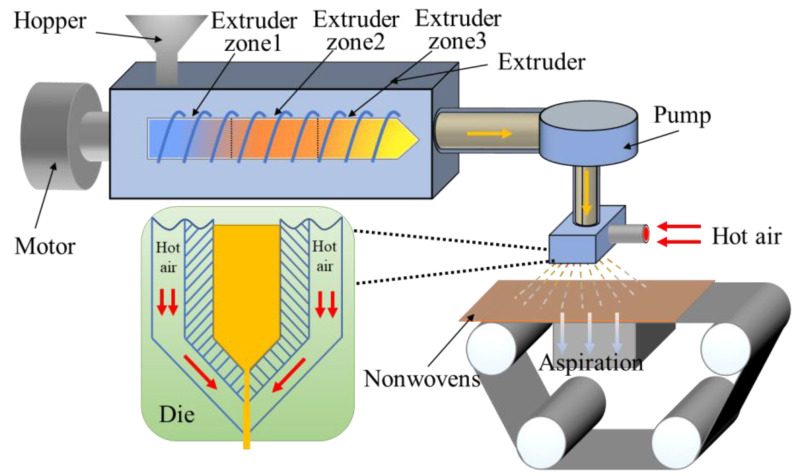
The process diagram shows the preparation of PLA/PBE micro-nanofiber fabrics via a melt-blown process.

The melt-blown process is shown in Figure 1. The PLA/PBE blended chips were added into the melt-blown equipment (MB-300, Suzhou Doro New Materials Technology Co., Ltd., Suzhou, China) through a hopper. The PLA/PBE blend polymer was melted into the extruder then ejected from the die using a pump. The ejected melt was drafted and cooled into micro-nanofibers using hot air. Finally, PLA/PBE micro-nanofibers form fabrics by self-adhesion under the action of indoor air. The main melt-blown process parameters are shown in Table 1.

### 2.3. Characters

**Figure 2:**
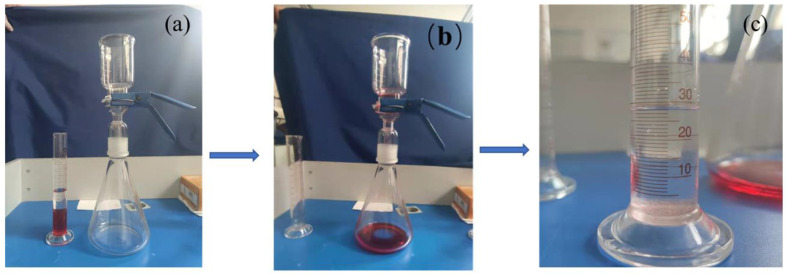
Oil/Water separation process: (**a**) self-made separation device; (**b**) filtering oil/water mixture. (**c**) volume of water after separation.

Thermogravimetric analysis of the PLA/PBE blended polymer was conducted with a thermal gravitational analysis system (TG 2019F1 Iris, NETZSCH Scientific Instruments Trading. CO. Ltd., Selb, Germany). The thermal properties of the PLA/PBE master batches were analyzed using a differential scanning calorimeter (TA DISCOVERY DSC25, Waters Corporation, Milford, MA, USA). The rheological properties of the PLA/PBE master batches were tested using a melt index tester (FBS-400C, Xiamen Forbus Testing Equipment Co., Ltd., Xiamen, China). The fiber diameters of the prepared samples were tested using scanning electron microscopy (Sigma 500, Zeiss, Germany). The strength properties of samples were tested using a mechanical tester (HD026S, Nantong Hongda Experimental Instrument Co., Ltd., Nantong, China) according to GB/T 24218.3-2010. The hydrostatic pressure of the samples was tested using a hydrostatic fabric tester (YG825G, Ningbo Textile Instrument Factory, Ningbo, China) according to GB/T 24218.16-2017. The water contact angle of the samples was tested using a contact angle system (SDC-350, Dongguan Shengding Precision Instrument Co., Ltd., Dongguan, China) according to standard DB44/T 1872-2016. The liquid/oil diffusion of the samples was expressed by the wetted area of soybean oil on the surface. The liquid climbing height of the samples was tested using a capillary effect tester according to standard FZ/T 01071-1999. The samples were weighed with an electronic balance. The filtration performance of the samples was determined with a self-made oil/water separation device as shown in Figure 2.

## 3. Results and Discussion

### 3.1. Properties of PLA/PBE Polymers

**Figure 3:**
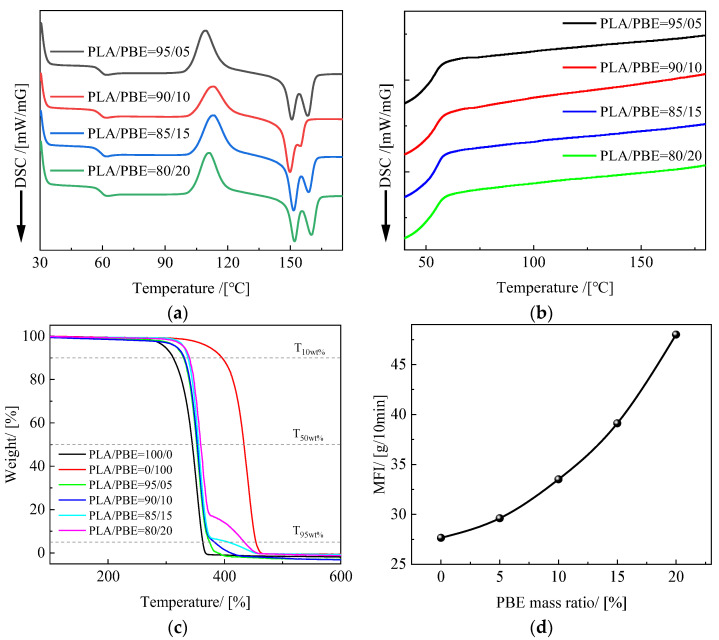
Thermal properties of PLA/PBE polymers: (**a**) heating curves of PLA/PBE polymers; (**b**) cooling curves of PLA/PBE polymers; (**c**) TG curves of PLA/PBE polymers; and (**d**) MFI curves of PLA/PBE polymers.

Figure 3a,b shows the crystallization and melting curves of PLA/PBE blended polymers with different PBE mass ratios. Obvious cold crystallization peaks during the second heating process can be seen in the figure at 60 °C and around 105 °C. Cold crystallization is typical in the PLA heating process. This is because the mobility of the PLA molecular chains increases with increasing temperature, which promotes the regular arrangement of molecular chains and leads to a crystalline rearrangement. The PLA melting peak exhibits two distinct peaks, which may be due to the melting and recrystallization of PLA, to the influence of the multilayer lamellae, or to the crystal structure [37]. The absence of a crystallization peak in the PLA cooling curve is due to the slow crystallization rate of PLA. The lack of an increase in either PBE’s heating or cooling curve is due to the absence of a crystallization zone in PBE and, therefore, the absence of a crystallization peak.

Figure 3c shows the thermogravimetric analysis (TG) curves for PLA/PBE blended polymers with different PBE mass fractions. The thermal decomposition process of both PLA and PBE polymers is a one-step reaction, as can be seen in the figure, with mass ratio thermal weight loss temperatures of 311 °C, 345 °C, and 362 °C for PLA at T10 wt%, T50 wt%, and T95 wt%, respectively. The mass ratio thermal weight loss temperatures of PBE at T10 wt%, T50 wt%, and T95 wt% are 396 °C, 433 °C, and 454 °C, respectively. Free radical theory suggests that the breakdown of PLA begins with the breaking of two CO bonds, which generates a variety of large free radicals centered on carbon and oxygen. These radicals can be further decomposed as they are split into smaller groups (CO, CO^2^, acetaldehyde, and methyl vinyl ketone), some of which undergo cyclization to produce propyl cross-esters by an additional elimination mechanism [38]. It was found that the T10 wt%, T50 wt%, and T95 wt% of the PLA/PBE blended polymer system all increased to varying degrees with the addition of PBE, and the decomposition temperature of the PLA/PBE blended polymer increased with the increase in the mass fraction of PBE, indicating that the addition of PBE increased the thermal stability of PLA.

Figure 3d shows the melt index curves for different PBE mass ratios at 220 °C. It can be seen from the graph that the melt flow index of PLA in this experiment was 27.7 g/10 min at 220 °C, the melt index of PBE was higher than that of PLA, and the melt index of PLA/PBE blended polymer increased with the increase in PBE mass ratios. This generally indicates that the polymer melt [39] has a good flow.

### 3.2. Morphological Structure and Fiber Diameter Distribution of PLA/PBE Micro-Nanofiber Fabrics

**Figure 4:**
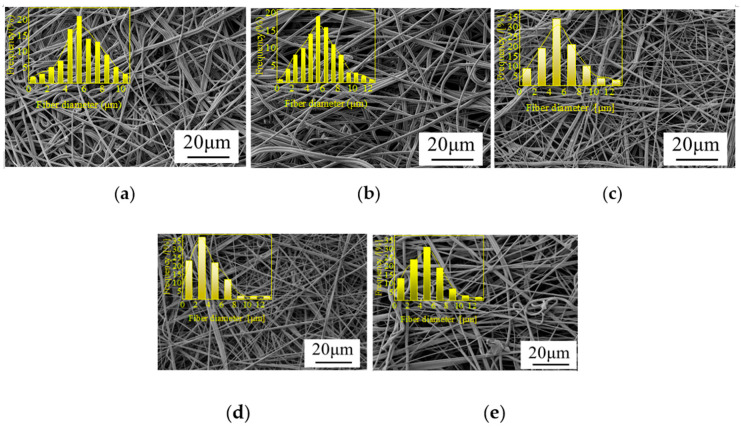
Scanning electron microscope (SEM) images and fiber diameter distributions on the surface of the fabrics with different PBE mass ratios: (**a**) PLA/PBE = 100/0 (**b**), PLA/PBE = 95/5, (**c**) PLA/PBE = 90/10, (**d**) PLA/PBE = 85/15, and (**e**) PLA/PBE = 80/20.

Figure 4 shows the morphological structure and fiber diameter distribution of PLA/PBE micro-nanofiber fabrics with different PBE blending ratios. It can be seen from the figure that the micro-nanofiber fabrics are composed of a three-dimensional mesh with a porous fiber structure with interconnected fibers. The diameter distribution is generally increased in the middle and lower at the ends, and the average fiber diameter changed less when the PBE mass ratio was 0–10%. The fiber diameter varied more when the PBE mass ratio was 10–20%. As can be seen in the fiber diameter distribution graph, the material fiber diameter 1–4 μm increased with the increase in PBE mass ratios. This may be due to the increased fluidity of the blended polymer melt caused by the plasticizing effect of PBE, which leads to a decrease in fiber fineness [40].

**Figure 5:**
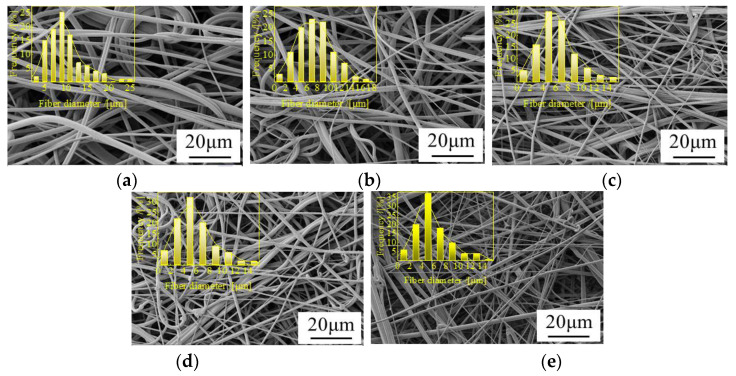
SEM images and fiber diameter distributions on the surface of the fabrics with different hot air pressures: (**a**) 0.01 MPa, (**b**) 0.02 MPa, (**c**) 0.03 MPa, (**d**) 0.04 MPa, and (**e**) 0.05 MPa.

From Figure 5, it can be seen that the fiber diameter decreased as the hot air pressure increased. The reasons for this may be related to the fact that the increase in hot air speed and hot air pressure make the PLA/PBE blended melt receive more drawing force and are thus drawn more fully. Hence, the fiber diameter decreased, and the fiber diameter distribution was more concentrated.

**Figure 6:**
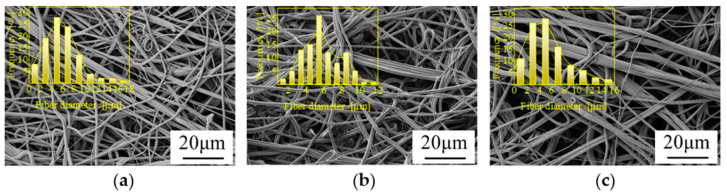

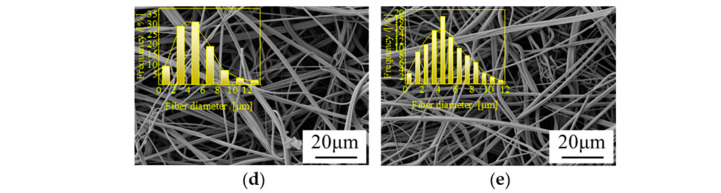
SEM images and fiber diameter distributions on the surface of the fabrics with different die-to-collector distance: (**a**) 10 cm, (**b**) 15 cm, (**c**) 20 cm, (**d**) 25 cm, and (**e**) 30 cm.

From Figure 6, it can be seen that the fiber diameter distributions of PLA/PBE micro-nanofiber fabrics with different die-to-collector distances have different characteristics, and the fiber diameter decreased with the increasing receiving distance. This may be because when the distance from the die head to the collector is low, the time of the melt under the action of hot air is shortened, and the fibers are prone to entanglement due to insufficient cooling, causing the silk phenomenon and the average diameter of the fiber to increase. When the die-to-collector distance is higher, the draw time of the melt in the hot air environment increases, and the fiber can be fully drafted and cooled. Hence, the average fineness of the fiber decreases, and because fiber “merging” decreases, the fiber diameter distribution becomes more uniform.

### 3.3. Mechanical Properties of PLA/PBE Micro-Nanofiber Fabrics

**Figure 7:**
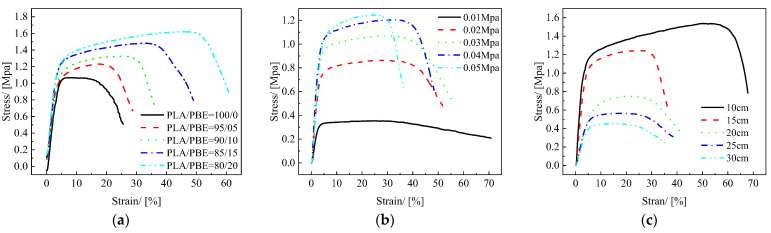
Mechanical properties of the fabrics: (**a**) stress/strain curves of different die-to-collector distances; (**b**) stress/strain curves of different hot air pressures; (**c**) stress/strain curves of different PBE ratios.

Oil/water separation materials need sufficient strength and toughness to support the oil/water mixture. From the stress/strain curves of PLA/PBE micro-nanofiber fabrics with different PBE mass ratios in Figure 7a, it can be seen that the strength of PLA/PBE micro-nanofiber fabrics increased with the increase in PBE mass ratios. The stress of PLA/PBE micro-nanofiber fabrics reached a maximum value of 1.63 MPa when the PBE mass ratio was 20% and increased about 40% compared to the stress of pure PLA micro-nanofiber1.09 MPa. The elongation at break of the PLA/PBE micro-nanofiber fabrics increased significantly with increasing PBE mass ratios. The elongation at break of PLA micro-nanofiber fabrics was only 6%, while the elongation at break of PLA/PBE micro-nanofiber fabrics reached 48% at 20% PBE mass ratios. Our analysis demonstrated that the average diameter of the material fibers decreased with the increase in the PBE mass ratios, so that the number of fibers of the same volume content increased, which resulted in more bonding points between fibers, causing tighter structures to form and increasing the strength of the PLA/PBE micro-nanofiber fabrics. The increase in the toughness of the PLA/PBE micro-nanofiber fabrics may be due to the interleaved entanglement of PBE and PLA in the blended polymer, which produces a blended composite system and increases the toughness of the blended mixed system due to the toughness of PBE.

The stress/strain curves of PLA/PBE micro-nanofiber fabrics with different hot air pressures are shown in Figure 7b. It can be seen that as the hot air pressure increased from 0.01 MPa to 0.05 MPa, the stress of the PLA/PBE micro-nanofiber fabric material increased from 0.3 MPa to 1.28 MPa, although its elongation at break did not change significantly. There are two possible reasons for this: on the one hand, it may be that the draft force increases with the increase in hot air pressure, enabling the fibers to draft more completely, resulting in a more acceptable fiber diameter and more accessible entanglement between fibers, thus increasing the bonding point between fibers; on the other hand, when the melt is rapidly drafted so that the orientation of macromolecules increases, which is conducive to the formation of fiber crystals, the strength of a single fiber increases, and this increases the tensile modulus of rupture. The small change in elongation at break may be because the change in extension at break is mainly related to the PBE mass ratios and less associated with the hot air pressure.

**Figure 8:**
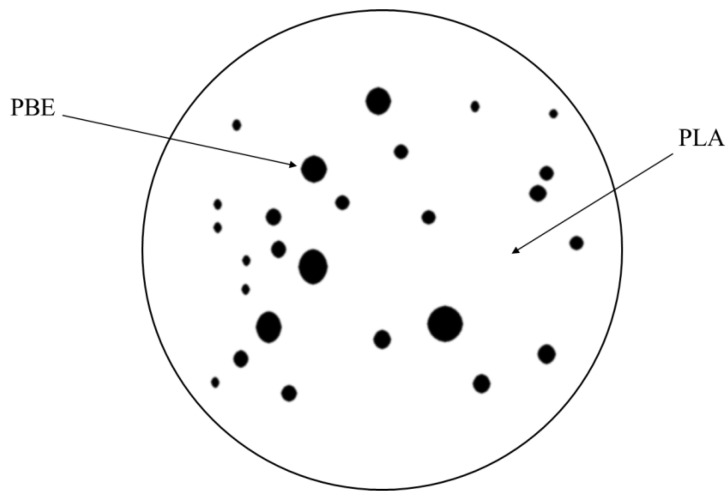
Schematic diagram of island structure.

Figure 7c shows the stress/strain curves of PLA/PBE micro-nanofiber fabrics at different receiving distances. The maximum stress value of PLA/PBE micro-nanofiber fabrics was 1.5 MPa, and the elongation at break was 51.1% when the receiving space was 10 cm; the minimum stress value was 0.43 MPa, and the elongation at break was 28.9% when the receiving distance was 30 cm. The reason for this may be that, although the melt can be fully drawn and cooled with the increase in the receiving space, the entanglement and bonding points between fibers increase, leading to an increase in the strength. Furthermore, the increase in the receiving distance will cause the temperature of the drafting hot air and the fiber tow to drop rapidly, resulting in a decrease in the thermal bonding efficiency between the fibers in the melt-blown fiber web, thus reducing the adhesion points per unit volume of the material, increasing bulk and thickness of the material. Therefore, the stress of the material decreases. Preliminary drawing of the melt allows the PBE to form an “island” structure with the PLA as shown in Figure 8, resulting in an increased material toughness. As the addition of PBE increases the fluidity of the PLA/PBE blend, as shown in the Figure 3d MFI, when the receiving distance increases, the fibers may break due to fiber overdrawing, which affects the strength and toughness of the material.

### 3.4. Hydrostatic Pressure Resistance of PLA/PBE Micro-Nanofiber Fabrics

**Figure 9:**
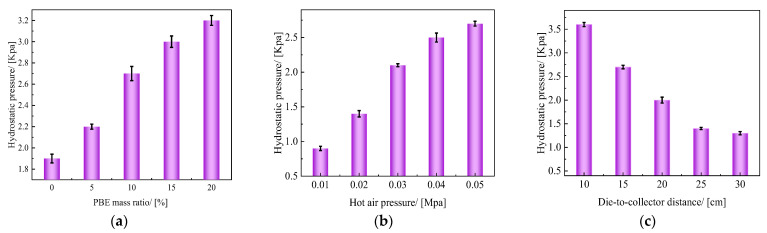
Hydrostatic pressure resistance of the fabrics: (**a**) different PBE mass ratios; (**b**) different hot air pressures; (**c**) different die-to-collector distances.

The hydrostatic pressure can indicate the strength of the water barrier of material [41], providing a basis for subsequent oil/water separation experiments. From Figure 9, it can be seen that the PBE mass ratios, hot air wind pressure, and receiving distance had significant effects on the hydrostatic pressure of the material. When the PBE ratio was 20%, the hydrostatic pressure resistance of the sample increased to 3.17 KPa. Moreover, the hydrostatic pressure of the sample increased from 0.865 KPa to 2.67 KPa when the hot air pressure was increased from 0.01 MPa to 0.05 MPa, and the hydrostatic pressure resistance of the sample decreased from 3.592 KPa to 1.142 KPa when the receiving distance increased from 10 cm to 30 cm. This may be because the increase in PBE mass ratios and hot air pressure increases the number of fibers with a diameter of less than 3 μm, and the smaller the pore size between fibers, the denser the material structure, the greater the pressure needed for water to pass through the sample, and the higher the hydrostatic pressure of the sample. The thickness of the material increases as the receiving distance increases, reducing its ability to block liquids due to the material being fluffier.

### 3.5. Water Contact Angle of PLA/PBE Micro-Nanofiber Fabrics

**Figure 10:**
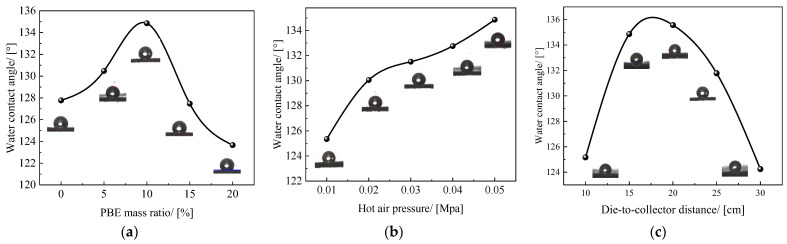
The water contact angle of the fabrics: (**a**) different PBE mass ratios; (**b**) different hot air pressures; (**c**) different die-to-collector distance.

The contact angles of PLA/PBE micro-nanofiber fabrics with different PBE mass ratios are shown in Figure 10a. It can be seen that the water contact angle of the samples increased when the PBE mass ratios were 0% to 10% and decreased when the PBE mass ratios were 10% to 20%. When the PBE mass ratio was 10%, the contact angle of the material reached a maximum of 134°. This is due to the increase in the melt-blown material fiber fineness in the PBE mass ratios range of 0% to 10%, which was demonstrated by the lower fiber diameter at the microscopic level and the increase in the fineness of the material surface at the macroscopic level. When the PBE mass ratio exceeded 10%, the contact angle decreased because the fiber diameter distribution of the material widened, resulting in an increase in the contact angle due to the appearance of “super coarse” fibers.

The contact angle of PLA/PBE micro-nanofiber fabrics with different hot air pressures is shown in Figure 10b. The contact angle of the material continuously increased with the increase in hot air pressure. The contact angle increased from 125° to 134° when the hot air pressure increased from 0.01 MPa to 0.05 MPa. This was probably due to the increase in the hot air pressure, the thinning of the fiber diameter due to the increase in the drafting force on the PLA/PBE melt, the appearance of a “rougher” material at the micro- and nanoscale, and the increase in the contact area between the liquid and the material, which led to an increase in the fabric’s contact angle according to the rough surface wetting model [42].

The contact angles of PLA/PBE micro-nanofiber fabrics with different DCDs are shown in Figure 10c. It can be found from the figure that the contact angle of the material first increases and then decreases with the increase in the receiving distance, and the change is large in the range of 10–15 cm and 20–30 cm, and the change is small in the range of 10–15 cm. It may be because when the receiving distance is in the range of 10–15 cm, the fibers become thinner and the surface smoothness decreases with the increase in the receiving distance, thereby increasing the contact angle of the material. Then with the further increase in the receiving distance, the thickness of the fiber web increases, and the fiber web becomes fluffy and not enough to support the gravity of the liquid, resulting in the liquid being trapped inside the material and the contact angle becomes smaller.

### 3.6. Liquid/Oil Diffusion of PLA/PBE Micro-Nanofiber Fabrics

**Figure 11:**
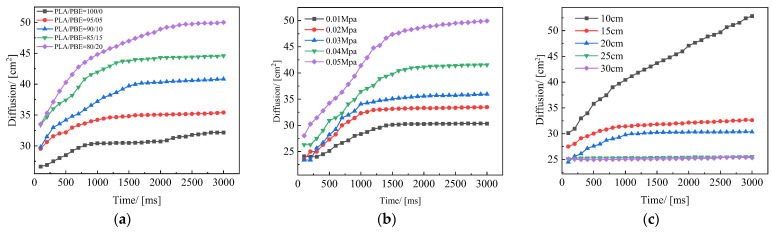
Liquid diffusion area of the fabrics: (**a**) different PBE mass ratios; (**b**) different die-to-collector distances; (**c**) different hot air pressures.

It can be seen from Figure 11a that the liquid diffusion areas of the samples with different PBE mass ratios all increased with time, but the speed of liquid diffusion was different in different periods, i.e., the liquid diffusion area increased rapidly in the beginning stage and then it increased at a slower rate. The reasons for this may be that liquid diffusion is more affected by the gravity of the liquid in the beginning stage, meaning that the fluid can quickly enter into the interior of the specimen, increasing the liquid diffusion speed. With the increase in the PBE mass ratio, the fiber fineness of the material decreases and the volume-to-surface area ratio is low, resulting in the enhancement of the material capillary force, causing the liquid diffusion area and diffusion speed to exhibit an increasing trend.

Figure 11b shows the variation curves of the liquid diffusion area of PLA/PBE micro-nanofiber fabric material with time for different hot air wind pressures. From the figure, it can be observed that the effect of the change in hot air pressure on the liquid diffusion rate in the beginning stage was not obvious, and the fluid diffusion rate remained approximately the same under different process conditions. In contrast, the maximum liquid diffusion area was 38cm^2^ when the air pressure was 0.04 MPa. This may be because with the increase in hot air pressure, the greater drafting force reduces the average fineness of the fibers, and the fabric thickness increases due to a more bulky structure. At the same time, the multi-scale effect of fiber diameter is also more significant [43], the reduction of the average fiber fineness makes the fiber have a larger specific surface area and stronger capillary force [44], so the rate of diffusion of the liquid is faster and the diffusion area of the liquid is larger.

The liquid diffusion curves of PLA/PBE micro-nanofiber fabrics with different receiving distances in Figure 11c show that the increase in the receiving distance led to a decrease in the fluid diffusion rate and a reduction in the diffusion area; thus, the material can store more liquid with the increase in receiving distance and the increase in porosity associated with an increase in the thickness of the material. When the receiving distance was greater than 25 cm, the liquid diffusion area of the fabric increased sharply for a shorter period. When the receiving distance was low, the liquid diffusion increased sharply for a more extended period of time, with the oil droplets on the surface of the sample quickly diffusing into the fabric and then rapidly diffusing along the surface of the fabric, thus increasing the wetting area and finally reaching a stable wetting area. This liquid diffusion behavior may arise from the coordination between two driving forces. On the one hand, the capillary forces provided by the surface area of the fibers drag the oil along the fibers; on the other, gravity easily exerts pressure on the oil droplets that have penetrated the material from the outside.

### 3.7. Liquid/Oil Wetting of PLA/PBE Micro-Nanofiber Fabrics

**Figure 12:**
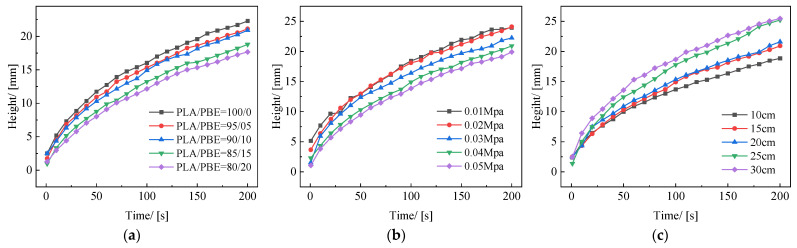
Liquid climbing height of the fabrics: (**a**) different PBE mass ratios; (**b**) different die-to-collector distance; (**c**) different hot air pressures.

**Figure 13:**
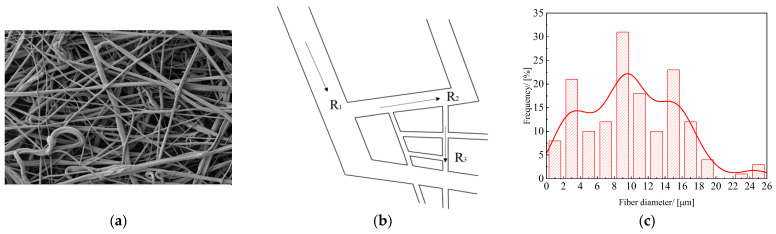
Tertiary branching structure: (**a**) SEM diagram of tertiary branch structure; (**b**) schematic diagram of the three-level branch structure, the fiber diameters: R_1_, R_2_, R_3_; (**c**) fiber diameter distribution of tertiary branched structure.

The variation curve of liquid wetting height with time for PLA/PBE micro-nanofiber fabrics with different PBE mass ratios can be seen in Figure 12a. The wetted area in the material varied with the core wicking time, which followed the typical core wicking behavior of porous fabrics, i.e., the increase in PBE increased the liquid climb height and climb speed; the material demonstrated the maximum climb height and speed when the PBE mass ratio was 20%. This may be because when the PBE mass ratio was 20%, the fiber diameter of melt-blown material had a tertiary branching structure as shown in Figure 13, which had a superior ability to store and transport liquid due to the higher Laplace Pressure [45]. Similarly, Zhang, H et al. [46] combined nonsolvent co-blending melt-blown technology with a functional bionic structure design to significantly improve the liquid transport properties of micro-nanofiber fabric materials by modulating the horizontal branching mesh structure of the fabrics.

Figure 12b shows the variation curves of the liquid climbing height of PLA/PBE micro-nanofiber fabric with different hot air pressures with time. The liquid climbing height of the material all increased with time, and the smaller the hot air pressure was, the faster the liquid climbed. Specifically, the material’s liquid capillary height increased at a faster rate in the initial stage (0~100 s) and the sample’s liquid capillary height increased more slowly in the stable stage (100~200 s). The possible reasons are that, in the initial stage of the capillary core suction, the liquid climbing height is small, the liquid gravity effect is small, and there are a large number of capillary pores in the sample to provide space for the liquid’s rapid climb. As the oil core suction proceeds, the height of the material’s capillary core suction increases, causing gravity to exert force on the liquid and the viscous force to gradually increase. At this point, the liquid core suction rise rate gradually stabilizes and reaches equilibrium.

Figure 12c shows the curves of PLA/PBE micro-nanofiber fabric with liquid climbing height as a function of time for different receiving distances. The liquid climbing height and speed both increased with the increase in receiving distance. Moreover, as the receiving distance increased, the fibers adhered, and when the receiving distance was 10 cm, they formed a highly filled mat. The fibers exhibited a curled and twisted morphology and the porosity increased as DCD increased to 30 cm. This structural change was attributed to two factors: the high-velocity air producing a higher lateral velocity with the increasing receiving distance, resulting in an irregular fiber draw, and the increasing receiving distance prolonging melting into fibers, which is necessary for polymer curing, resulting in increased fiber fineness and an increased porosity and oil vertical penetration rate.

### 3.8. Oil/Water Separation Efficiency of PLA/PBE Micro-Nanofiber Fabrics

The oil absorption performance of the micro-nanofiber fabrics is expressed by calculating the oil absorption multiple. As can be seen in Table 2, the oil absorption multiplier of the PLA/PBE micro-nanofiber fabric increased from 4.65 to 6.22 when the PBE mass ratio increased from 0% to 20%. The oil absorption multiplier of the PLA/PBE micro-nanofiber fabric rose from 4.69 to 5.87 when the hot air pressure decreased from 0.05 MPa to 0.01 MPa. The oil absorption multiplier of the PLA/PBE micro-nanofiber fabric rose from 4.13 to 10.34 when the receiving distance increased from 10 cm to 30 cm. From our analysis in 3.6, it can be seen that the fiber fineness of the material decreases, and the volume surface area ratio decreases with the increase in the PBE mass ratio, which leads to the enhancement of the material capillary force, so the oil absorption performance is improved. From the authors’ analysis in 3.7, it can be seen that the increase in the receiving distance will also lead to the increase in fiber fineness, porosity, and vertical oil permeability, thereby improving the oil absorption performance. While the hot air pressure has little effect on the oil absorption properties.

**Figure 14:**
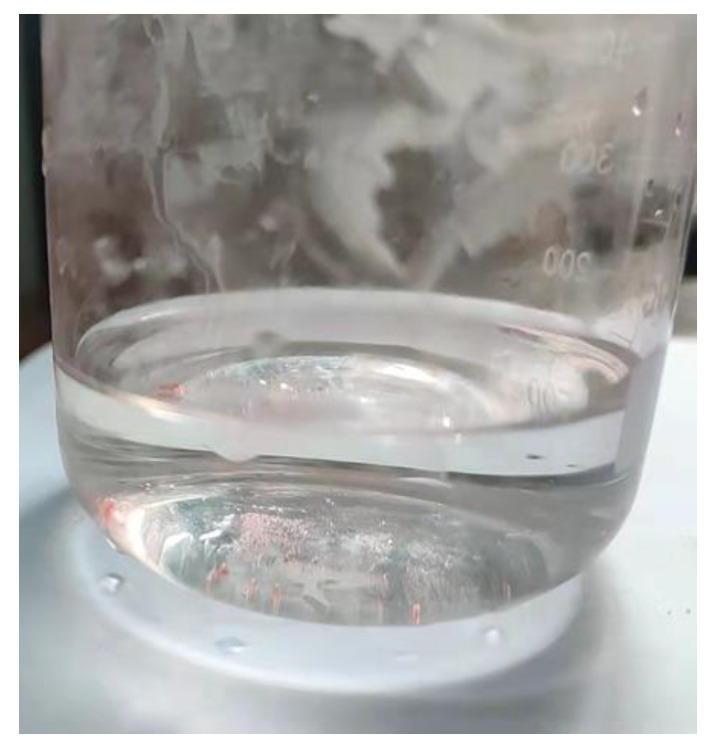
Result of oil-water separation.

The oil/water separation material has specific requirements regarding strength due to the need to bear the weight of the liquid. In contrast, the receiving distance and hot air wind pressure have a significant impact on the mechanical properties of PLA/PBE micro-nanofiber fabrics, i.e., insufficient hot air pressure combined with too large of a received distance results in insufficient power. Therefore, only the oil/water separation experimental process was performed for micro-nanofiber fabrics with different PBE mass ratios. The experimental results are shown in Figure 14. The observed phenomena demonstrate that the PLA/PBE micro-nanofiber fabrics with different PBE mass ratios all have excellent oil/water separation efficiency by gravity alone due to their excellent lipophilic hydrophobicity.

## 4. Conclusions

In this work, PLA/PBE polymers were prepared by blending and melting, and the effect of PBE addition on the thermal stability of PLA was analyzed. The melt-blown process was used to successfully prepare PLA/PBE micro-nanofiber fabric with hydrophobic and lipophilic properties. The effects of the PBE content and melt-blown process on the fabric structure and properties were investigated. The results show that the addition of PBE improves the thermal stability of PLA. The PLA/PBE micro-nanofiber fabrics demonstrated significantly increased toughness and strength as compared with the PLA micro-nanofiber fabrics. The strength and elongation at break of the PLA/PBE micro-nanofiber fabrics increased by 255% and 455%, respectively. Moreover, when the PLA/PBE mass ratio was 80/20.PLA/PBE, the micro-nanofiber fabrics were hydrophobic, and variations in the process allowed the contact angle of the material to be adjusted in the range 123°–136°. The maximum oil absorption rate of PLA/PBE micro-nanofiber fabrics was 10.34, demonstrating their excellent oil/water separation performance. The prepared micro-nanofiber fabric is suitable for industrial production and can reduce oil pollution by efficiently separating oil/water mixtures.

## Figures and Tables

**Table 1:** The main parameters of the melt-blown process.

Motor Speed	Extruder Zone1 Temperature	Extruder Zone2 Temperature	Extruder Zone3 Temperature	Pump Temperature	Die Temperature	Hot Air Temperature	Pump Speed
4 Hz	180 °C	200 °C	220 °C	220 °C	220 °C	230 °C	5 Hz

**Table 2:** The oil absorption rate of the prepared samples.

PLA/PBE	DCD/(cm)	Hot Air Pressure/[MPa]	Oil Absorption
100/0	15	0.05	4.64
95/5	15	0.05	4.83
90/10	15	0.05	4.69
85/15	15	0.05	5.96
80/20	15	0.05	6.22
90/10	10	0.05	4.22
90/10	20	0.05	7.27
90/10	25	0.05	9.32
90/10	30	0.05	10.34
90/10	15	0.01	5.87
90/10	15	0.02	5.82
90/10	15	0.03	5.32
90/10	15	0.04	5.22

## Data Availability

The data presented in this study are available on request from the corresponding author.
